# DIFC-Net: Diffusion-Intrinsic Feature Capture for AI-Generated Image Detection

**DOI:** 10.3390/s26082389

**Published:** 2026-04-13

**Authors:** Shaofeng Lu, Jin Tian, Yujin Zhang, Fei Wu, Li Gong, Han Pan

**Affiliations:** 1 School of Electric and Electronic Engineering, Shanghai University of Engineering Science, Shanghai 201620, China; m320123321@sues.edu.cn (S.L.); yjzhang@sues.edu.cn (Y.Z.); wufei@sues.edu.cn (F.W.); 2 School of Geographic Sciences, East China Normal University, Shanghai 200241, China; lgong@re.ecnu.edu.cn; 3 School of Aeronautics and Astronautics, Shanghai Jiao Tong University, Shanghai 200240, China; hanpan@sjtu.edu.cn

**Keywords:** diffusion model detection, image forensic analysis, latent diffusion inversion, residual discrepancy, multimodal feature fusion, synthetic image authenticity

## Abstract

Diffusion models (e.g., Stable Diffusion, DALL·E 3) can now generate images that are nearly indistinguishable from real ones, making synthetic image detection increasingly challenging. We propose DIFC-Net, a diffusion-intrinsic detection framework that identifies AI-generated images by analyzing their reconstruction behavior during diffusion inversion rather than relying on visual artifacts. DIFC-Net jointly captures spatial discrepancy signals and latent diffusion trajectory evolution, and adaptively fuses them into a unified forensic representation. Extensive cross-model evaluations show that DIFC-Net achieves 90.29% average AUC on multiple unseen diffusion generators, outperforming state-of-the-art detectors while maintaining strong generalization without relying on training-time knowledge of specific generative models.

## 1. Introduction

Diffusion-based generative models, such as SDXL, Stable Diffusion, and DALL·E 3, have made significant advancements in synthesizing images with high perceptual realism and semantic coherence [1]. While these models enable creative applications and large-scale data augmentation, they also pose a significant challenge to multimedia forensics by making it difficult to detect synthetic content. These images, although visually indistinguishable from real ones, have the potential to contaminate datasets, mislead downstream vision systems, and erode public trust in automated decision-making.

Traditional image detectors, originally developed for GAN-based models, have been shown to struggle when faced with today’s high-fidelity diffusion outputs, especially under distribution shift [2,3]. Most existing methods rely either on handcrafted features, such as frequency or noise statistics [4], which are vulnerable to common post-processing techniques, or on supervised classifiers (CNNs or Transformers) [5,6] that tend to overfit appearance patterns and lack generalization to unseen generators [7].

A fundamental limitation of these approaches is their treatment of image detection as a static problem, focusing on visual artifacts or pixel-level differences. In contrast, diffusion-based synthesis occurs through an iterative denoising process in latent space [8,9]. This process allows real images to reconstruct with stable, manifold-consistent dynamics, while synthetic images tend to exhibit irregular transitions and inconsistent residual structures [10]. These observations suggest that a more effective approach to detection would involve probing the reconstruction behavior of images under diffusion inversion, rather than relying solely on final pixel appearance.

To address these limitations, we propose DIFC-Net, a novel detection framework that leverages the intrinsic behavior of diffusion processes. DIFC-Net captures both spatial discrepancy signals from reconstruction residuals and temporal evolution patterns along the latent diffusion trajectory. Our architecture consists of three main modules: the Visual Comparator Path (VCP), which utilizes channel–spatial attention and cross-modal attention to extract informative residual features; the Trajectory Feature Model (TFM), which models the latent dynamics of diffusion through multi-head self-attention; and the Active Capture Fusion (ACF) module, which adaptively integrates visual and temporal cues through a learnable gating mechanism to form a robust forensic representation.

In summary, the main contributions of this work are:

- We propose DIFC-Net, a novel diffusion-intrinsic detection framework that identifies AI-generated images by analyzing their reconstruction behavior during diffusion inversion, rather than relying on static pixel-level artifacts.
- We introduce a unified multi-path learning architecture that integrates spatial residual discrepancies and latent diffusion trajectory evolution, enabling robust detection of generative inconsistencies across diverse models.
- We demonstrate strong cross-model generalization and interpretability, achieving state-of-the-art performance on unseen diffusion models and providing transparent forensic reasoning through residual visualization and attention heatmaps.

## 2. Related Work

In recent years, distinguishing real images from synthetic images generated by generative models (GAN/Diffusion Model) has become an important research direction in computer vision and multimedia forensics. Research [11] on this issue can be roughly divided into five main lines: (i) low-level artifacts and frequency domain signals; (ii) end-to-end detection based on deep features; (iii) detection and reconstruction consistency of diffusion model specificity; (iv) fingerprinting, attribution, and traceability; (v) robustness, generalizability, and calibration.

### 2.1. Low-Level Artifacts and Frequency Domain Signals

Early methods start from “interpretable physical or numerical clues” in the image formation process, focusing on systematic artifacts left over from upsampling, deconvolution, interpolation kernels, color statistics, and other aspects. For example, the spectral energy distribution differences induced by upsampling are used as stable evidence for GAN products [5]; high-frequency response and energy decay patterns [4] can also distinguish between real and synthetic images. In the face scene, FaceForensics++ [12] has constructed a standard benchmark and shown that JPEG compression and resampling can significantly affect frequency domain clues. Such methods are interpretable and computationally economical, but their accuracy tends to degrade when crossing generators, strong post-processing, or out-of-domain distributions [6]. In the specific modeling of the frequency domain, two-dimensional DFT/DCT [3,5,13] spectral replication and harmonic artifacts are often extracted and input into shallow classifiers or lightweight CNNs for rapid detection.

### 2.2. End-to-End Detection Based on Deep Features

To overcome the fragility of handcrafted cues, end-to-end CNN/Transformer has gradually become mainstream. Systematic studies [6] have shown that the current stage of CNN-generated images are generally detectable, and evaluated the robustness under real-world perturbations such as JPEG compression and blurring. From an interpretable perspective, convolutional traces and texture statistics can serve as a bridge for interpretable deep evidence [13]; from a forensic perspective, detection and manipulation recognition/consistency verification are integrated processes [14]. In recent years, an important branch has been the use of pretrained visual/multimodal models as backbones, such as CLIP/ViT, MAE, and BLIP2, which are efficiently fine-tuned through parameters such as LoRA to improve generalization ability [15,16,17,18]. At the same time, incremental learning, distillation, transfer, and ensemble have shown positive performance in improving cross-domain stability [2,19,20,21,22,23].

### 2.3. Detection and Reconstruction Consistency of Diffusion Model Specificity

After the rise of diffusion models, such as DDPM, Stable Diffusion, and SDXL, the focus of AI-generated image detection has gradually shifted from conventional GAN artifacts to the consistency and reversibility of diffusion processes. In particular, DDIM and related methods substantially reduce reconstruction steps while preserving reversibility [7], and diffusion models have also been systematically analyzed in terms of generation quality and stability [24]. A representative line of recent studies emphasizes inversion–reconstruction alignment in the diffusion latent space, where systematic differences between real and synthetic images can be revealed through residual inconsistency, reconstruction error, or latent mismatch. Representative examples include DNF [10], DIRE/DistilDIRE/LaRE^2^ [8,19], SeDID [25], and FIRE [26], which mainly exploit reconstruction errors or noise residuals; FakeInversion and DRCT [9,27], which fuse original images, decoded noise, and reconstructions into multi-view inputs; TOFE [28], which introduces text-guided inversion optimization; and DIP [29,30], which builds source fingerprints from reconstruction consistency. Recent studies have also highlighted broader evaluation dimensions for AI-generated image detection, including generalization, robustness, and explainability [31], while diffusion-aligned detection has further explored timestep-aware evidence extracted from iterative noising and denoising processes [23].

Despite these advances, most existing diffusion-oriented methods still rely primarily on a single dominant cue, such as reconstruction error, residual inconsistency, multi-view inversion input, or reconstruction consistency fingerprints. By contrast, DIFC-Net is designed to jointly capture two complementary types of diffusion-intrinsic evidence: fine-grained discrepancy reasoning over the reconstruction triplet and timestep-wise latent trajectory evolution during diffusion inversion. To better position DIFC-Net with respect to existing detectors and the experimental baselines used in this study, Table 1 summarizes the methodological differences among representative AI-generated image detection methods and DIFC-Net.

### 2.4. Fingerprint, Attribution and Traceability

Compared with “true or false classification”, attribution/traceability emphasizes the identification of specific generators or training sources. Some research work has shown that generators with different training/architectures will leave identifiable fingerprints in their outputs [42,43]; ideas such as Noiseprint [44] and other “source fingerprints” are also instructive for generator attribution. At the industry level, content certificates/watermarks (C2PA, Stable Signature, Tree-Ring, etc.) provide active traceability methods; when watermarks are missing or tampered with, passive forensics remains the security bottom line [45,46].

### 2.5. Datasets, Robustness and Calibration

In terms of benchmarks, FaceForensics++, GenImage, and SyntheBusters [1,12,47] cover multiple generators and scenarios, which is helpful for quantifying cross-domain generalization. In terms of robustness, current research shows that compression/resampling, geometric editing, and adversarial perturbations have a significant impact on detectors, which can be effectively mitigated through structural enhancement and frequency domain robust regularization [9,48]. In addition, probability calibration is key to practical deployment: modern deep models often exhibit “overconfidence”, and it is necessary to combine metrics such as ECE/Brier with temperature scaling, label smoothing, and other methods to improve reliability [49,50].

## 3. Methods

**Figure 1:**
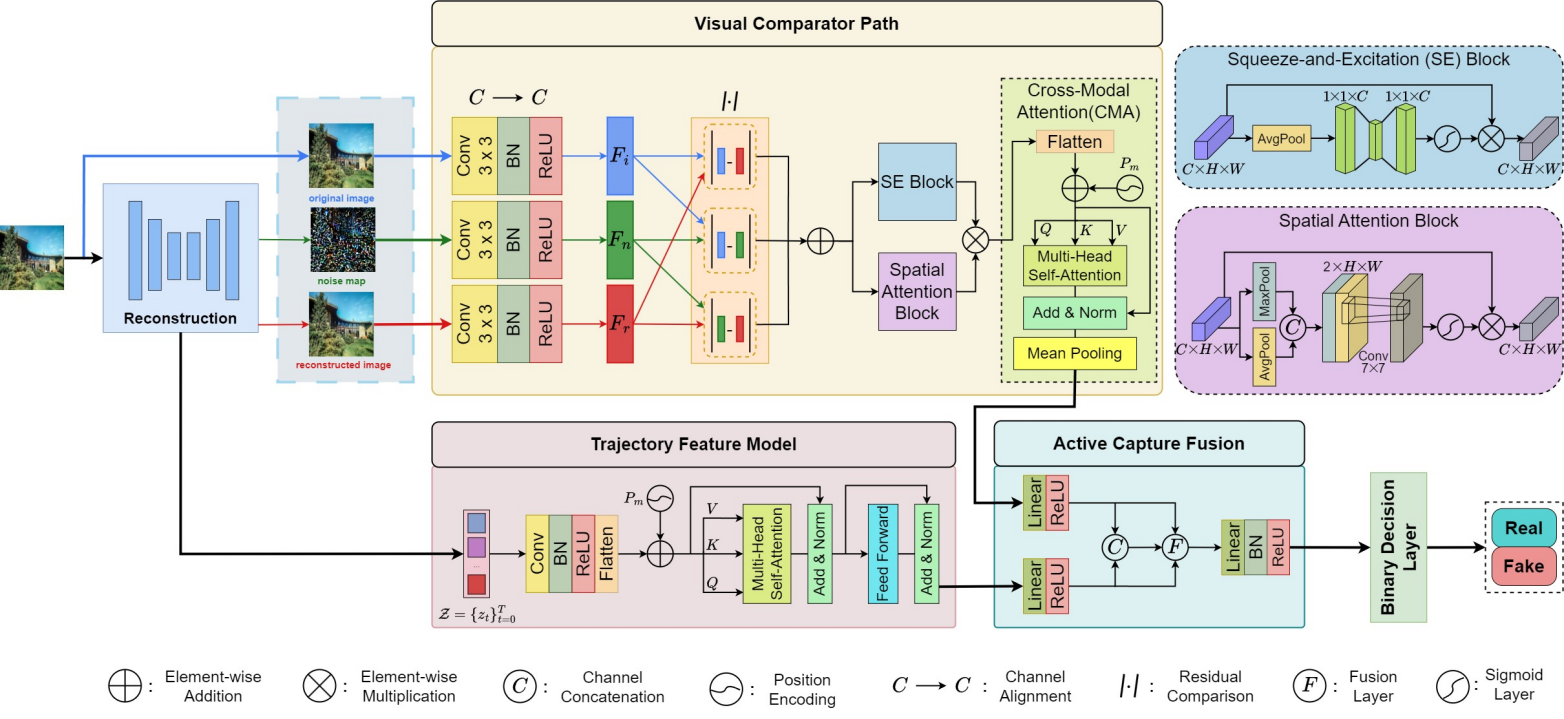
Overall framework of DIFC-Net. The reconstruction module performs diffusion inversion to generate a triplet $\left\{I,I_{n},I_{r}\right\}$. The Visual Comparator Path (VCP) extracts spatial cues through feature projection, residual differences, and attention mechanisms. The Trajectory Feature Model (TFM) models temporal evolution using tokenized latent trajectories and multi-head self-attention. The Active Capture Fusion (ACF) module fuses VCP and TFM features with a gating mechanism for final classification.

The proposed DIFC-Net detects diffusion-generated images by leveraging both reconstruction inconsistencies and latent trajectory cues. Given an input image, the reconstruction module performs diffusion inversion, producing multiple complementary views—original, reconstructed, and noise-perturbed images—that capture both visual and latent discrepancies. The Visual Comparator Path (VCP) focuses on fine-grained spatial differences among these views, using attention mechanisms to highlight subtle inconsistencies introduced during synthesis. Meanwhile, the Trajectory Feature Model (TFM) models the latent diffusion trajectory and captures its temporal evolution through multi-head self-attention. The outputs from VCP and TFM are then fused by the Active Capture Fusion (ACF) module, which adaptively integrates spatial and temporal information into a unified feature representation. Finally, a lightweight classifier performs the real/fake prediction. This multi-branch design enables DIFC-Net to reason jointly over spatial discrepancies and latent diffusion dynamics, as illustrated in Figure 1.

### 3.1. Reconstruction

**Figure 2:**
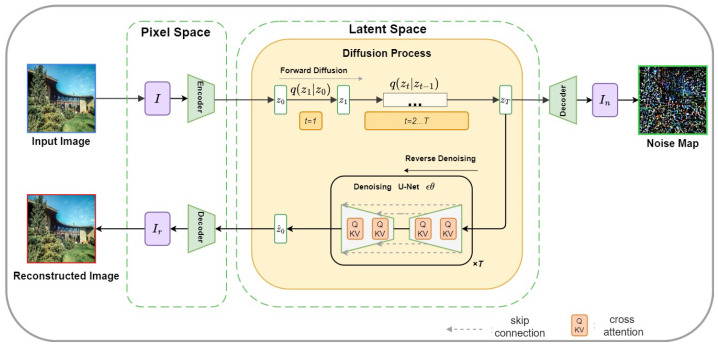
Reconstruction module using diffusion inversion. The input image is encoded into latent representation $Z_{0}$. During forward diffusion, latent states ${\left\{Z_{t}\right\}}_{t=1}^{T}$ are perturbed, and in reverse denoising, the model predicts noise using a U-Net with cross-attention layers to recover the latent representation. The decoded latent produces the reconstructed image $I_{r}$ and predicted noise map $I_{n}$, forming the reconstruction triplet $\left\{I,I_{n},I_{r}\right\}$ and providing explicit cues for visual discrepancy learning.

The reconstruction module processes the input image through the forward and reverse diffusion stages. As illustrated in Figure 2, the latent representation is first generated by a pretrained VAE encoder, then progressively perturbed during the forward diffusion process. In the reverse diffusion process, the U-Net model predicts the noise component and refines the latent representation at each step. The decoded latent representation yields both the reconstructed image and the noise map, which together form the reconstruction triplet. This process provides explicit cues for visual discrepancy learning.

#### 3.1.1. Forward Diffusion

To simulate the gradual degradation of visual information, Gaussian noise is progressively injected into the latent representation during the forward diffusion process. Given an input image I, it is first encoded into a compact latent representation $Z_{0}=E\left(I\right)$, where $Z_{0}\in R^{4\times \frac{H}{8}\times \frac{W}{8}}$. This latent state then undergoes a series of perturbations:

(1) $$q\left(Z_{t}|Z_{t-1}\right)=\sqrt{1-\beta_{t}}\;Z_{t-1}+\sqrt{\beta_{t}}\;\epsilon_{t},\;\epsilon_{t}∼N\left(0,I_{d}\right),$$

where $\beta_{t}$ denotes the noise variance schedule controlling the degradation rate at step *t*. The forward diffusion process continues until timestep *T*, resulting in the maximally noised latent state $Z_{T}$. The decoder D(·) is then applied to this latent variable to generate the noise map: $I_{n}=D\left(Z_{T}\right)$, which visualizes the stochastic, structure-free residuals in the image, providing DIFC-Net with explicit cues for learning visual discrepancies between real and synthetic content.

#### 3.1.2. Reverse Denoising Process

To recover the original content, DIFC-Net performs reverse diffusion using a denoising U-Net $\epsilon_{\theta }\left(Z_{t},t\right)$. This U-Net contains symmetric downsampling and upsampling blocks connected through residual skip links and cross-attention layers (QKV projections). The denoising process iteratively refines the latent representation by predicting the noise component at each timestep and updating the latent state as follows:

(2) $$\mu_{\theta }\left(Z_{t},t\right)=\frac{1}{\sqrt{\alpha_{t}}}\left(Z_{t}-\frac{\beta_{t}}{\sqrt{1-{\bar{\alpha }}_{t}}}\epsilon_{\theta }\left(Z_{t},t\right)\right),\;Z_{t-1}=\mu_{\theta }\left(Z_{t},t\right)+\sigma_{t}\eta ,$$

where $\alpha_{t}=1-\beta_{t}$ controls per-step signal preservation, $\beta_{t}$ is the noise variance, and $\sigma_{t}$ determines the stochasticity of sampling. This iterative process continues from t=T down to t=1, with the final latent state ${\hat{Z}}_{0}$ being decoded by the VAE decoder to obtain the reconstructed image $I_{r}=D\left({\hat{Z}}_{0}\right)$, where $I_{r}$ is the reconstructed version of the original image, now restored from the noisy latent space.

#### 3.1.3. Triplet and Latent Trajectory

The reconstruction process yields a triplet consisting of the original image I, the reconstructed image $I_{r}$, and the noise map $I_{n}$:$\left\{I,I_{n},I_{r}\right\}=\left\{I,D\left(Z_{T}\right),D\left({\hat{Z}}_{0}\right)\right\}$, which represents the original image, the noise-perturbed image, and the denoised reconstruction, respectively. Additionally, the latent diffusion trajectory $Z={\left\{Z_{t}\right\}}_{t=0}^{T}$ records the evolution path of the image from corrupted noise to semantically coherent content. This trajectory captures the temporal dynamics of the diffusion process, providing additional cues that are utilized in subsequent modules (Section 3.2 and Section 3.3) to further enhance the model’s ability to distinguish between real and synthetic images.

### 3.2. Visual Comparator Path

The Visual Comparator Path (VCP) aims to extract discriminative residual cues by explicitly comparing the feature maps obtained from the reconstruction triplet $\left(I,I_{n},I_{r}\right)$. Each image is first passed through a shared convolutional projection backbone consisting of Conv–BN–ReLU layers, which ensures consistent feature extraction and suppresses encoder bias. This shared backbone yields the feature representations $F_{\mathrm{img}},F_{\mathrm{recon}},F_{\mathrm{noise}}\in R^{C\times H\times W}$, each normalized to stabilize feature magnitude and prevent local illumination differences from dominating the comparison.

To reveal fine-grained inconsistencies, we compute element-wise absolute differences across each feature pair:

(3) $$D_{ir}=\left|F_{img}-F_{recon}\right|,\;D_{in}=\left|F_{img}-F_{noise}\right|,\;D_{rn}=\left|F_{recon}-F_{noise}\right|,$$

here, $D_{ir}$ highlights reconstruction deviation, capturing areas where structural recovery is imperfect; $D_{in}$ reflects noise perturbation, showing regions sensitive to diffusion noise; and $D_{rn}$ models reconstruction noise bias, indicating areas where the model overfits residual artifacts. Using absolute differences ensures symmetry and robustness against scaling variations, while shared normalization maintains invariance to global intensity shifts.

The three discrepancy maps are then fused into a unified residual tensor through learnable aggregation:

(4) $$R=\alpha D_{ir}+\beta D_{in}+\gamma D_{rn},\;\alpha ,\beta ,\gamma \geq 0,\;\alpha +\beta +\gamma =1,$$

here, α, β, and γ control the relative contribution of each discrepancy source. During training, the model automatically adjusts these weights, assigning higher importance to texture-sensitive terms (e.g., $D_{rn}$) when artifacts appear in high-frequency regions and to structural terms (e.g., $D_{ir}$) when distortion occurs in smooth areas.

To emphasize informative residual regions, VCP applies a channel–spatial attention refinement:

(5) $$A=\sigma \left(SE\left(R\right)\right)⊙\sigma \left({\mathrm{SpatialConv}}_{7\times 7}\left(R\right)\right),\;F_{r}^{\prime }=A⊙R,$$

where σ(·) denotes the sigmoid activation and ⊙ represents element-wise multiplication. The channel branch (SE) adaptively reweights each feature channel according to its global residual salience, while the spatial 7×7 convolution branch localizes where strong inconsistencies occur (e.g., object boundaries or repeated textures). A subsequent 1×1 convolutional bottleneck further mixes and normalizes the attended features without altering the residual semantics.

To capture cross-modal dependencies and long-range contextual relationships among the refined residual features, we introduce a Cross-Modal Attention (CMA) module as the final stage of the Visual Comparator Path (VCP) (Figure 1). The CMA module functions as a Transformer-based comparator. The refined residual feature $F_{r}^{\prime }$ is first flattened into a token sequence and augmented with positional encoding. This token sequence is then processed by multi-head self-attention (MHA) to capture global residual interactions across all positions and modalities. Finally, the output is normalized and pooled to obtain a compact visual comparator vector $v_{\mathrm{cmp}}$. Formally, the cross-modal attention operation is expressed as:

(6) $$Z^{\prime }=\mathrm{MHA}\left(Z_{p},Z_{p},Z_{p}\right),$$

where $Z_{p}=\mathrm{Flatten}\left(F_{r}^{\prime }\right)+P_{pos}$ denotes the flattened feature with added positional encoding $P_{\mathrm{pos}}$ to preserve spatial relationships. The resulting embedding $v_{cmp}$ is the context-aware residual descriptor that aggregates both intra-modal and inter-modal residual cues. Instead of relying solely on localized residual differences, CMA aggregates these discrepancies globally, producing a semantically aligned representation. This fused vector is then passed to the Active Capture Fusion (ACF) module for final reasoning with trajectory features.

### 3.3. Trajectory Feature Model

The Trajectory Feature Model (TFM) captures the temporal evolution of latent representations generated during diffusion inversion. Instead of relying solely on static spatial discrepancies, TFM models how the latent representation changes over diffusion timesteps, enabling DIFC-Net to exploit dynamic reconstruction behavior. Given a latent trajectory $Z={\left\{Z_{t}\right\}}_{t=0}^{T}$, each latent state $Z_{t}\in R^{C_{z}\times H_{z}\times W_{z}}$ corresponds to a diffusion timestep.

Each latent tensor is first transformed into a compact spatial representation $H_{t}\in R^{C\times H^{\prime }\times W^{\prime }}$, which is then flattened into a token matrix $X_{t}\in R^{N\times C}$ and augmented with positional encoding $P_{t}$.

To normalize channel statistics and suppress high-frequency noise, each latent $Z_{t}$ is first passed through a lightweight convolutional stem, producing a compact spatial representation $H_{t}$, where $H_{t}\in R^{C\times H^{\prime }\times W^{\prime }}$ and *C* denotes the embedding width. This feature map $H_{t}$ is then flattened into a sequence of $N=H^{\prime }\times W^{\prime }$ spatial tokens and augmented with a learnable positional encoding, forming the token representation $X_{t}\in R^{N\times C}$. The positional encoding $P_{t}$ embeds both spatial layout and temporal order, ensuring that the diffusion-step dependency is preserved.

All token sequences ${\left\{X_{t}\right\}}_{t=0}^{T}$ are concatenated and processed using multi-head self-attention, enabling the model to learn global relationships and temporal consistency across the entire latent trajectory. This operation produces an updated representation $Z^{\prime }$, where attention allows informative diffusion steps to influence others. The result then passes through a position-wise feed-forward refinement with residual connections and normalization, yielding $Z^{\prime \prime }$, a temporally enhanced latent representation.

Finally, global mean pooling is applied over all tokens across all timesteps, producing the trajectory descriptor $f_{traj}\in R^{C}$. This compact vector summarizes how latent states evolve from noise to semantically stable structures throughout the reconstruction process.

The resulting trajectory feature $f_{traj}$ provides temporal context that complements the spatial discrepancy embedding $v_{cmp}$ obtained from the Visual Comparator Path. By jointly capturing diffusion dynamics and reconstruction inconsistencies, TFM enables DIFC-Net to detect subtle generative traces that static spatial methods may fail to observe.

### 3.4. Active Capture Fusion

The Active Capture Fusion (ACF) module integrates the spatial discrepancy vector $v_{cmp}$ obtained from the Visual Comparator Path and the temporal evolution vector $f_{traj}$ derived from the Trajectory Feature Model, enabling DIFC-Net to reason jointly across heterogeneous feature domains. While $v_{cmp}\in R^{C_{v}}$ encodes reconstruction inconsistencies in pixel space, $f_{traj}\in R^{C_{t}}$ captures latent diffusion dynamics over time.

Before fusion, both feature vectors are projected into a shared latent space to ensure consistent dimensionality:

(7) $$v^{\prime }=W_{v}v_{cmp}+b_{v},\;f^{\prime }=W_{f}f_{traj}+b_{f},$$

where $W_{v}\in R^{C\times C_{v}}$ and $W_{f}\in R^{C\times C_{t}}$ are linear projections that can be learned and $b_{v}$, $b_{f}$ are bias terms.

To adaptively balance the contribution of visual and trajectory modalities, the Active Capture Fusion (ACF) module introduces a learnable gating mechanism. The projected visual feature $v^{\prime }$ and trajectory feature $f^{\prime }$ are first concatenated and passed through a linear transformation followed by a sigmoid activation to produce channel-wise gating coefficients. The gated fusion is then computed as:

(8) $$g=\sigma \;\left(W_{g}\left[v^{\prime };f^{\prime }\right]+b_{g}\right),\;f_{fusion}=g⊙v^{\prime }+\left(1-g\right)⊙f^{\prime },$$

where $W_{g}$ is the learnable projection matrix that maps the concatenated feature vector into channel-wise importance weights, and $b_{g}$ is the learnable bias term. The gating vector $g\in {\left[0,1\right]}^{C}$ determines, for each channel, how much emphasis should be placed on the visual discrepancy representation $v^{\prime }$ versus the temporal trajectory feature $f^{\prime }$. This adaptive modulation enables ACF to dynamically highlight visual cues when spatial inconsistency is strong and rely more on trajectory patterns when temporal diffusion signals are more discriminative.

The fused representation is further refined through a lightweight transformation block:

(9) $$f_{acf}=\delta \;\left(\mathrm{BN}(W_{c}f_{fusion}+b_{c})\right),$$

where $W_{c}\in R^{C\times C}$ is a learnable linear mapping, and BN(·) denotes batch normalization.

Through this adaptive gating and nonlinear refinement, the ACF module actively balances contributions from visual and temporal modalities, achieving a synergistic fusion that improves robustness under diverse diffusion artifacts. As illustrated in Figure 1, it serves as the convergence point of DIFC-Net, enabling holistic discrimination by integrating spatial attention, trajectory evolution, and adaptive modality weighting.

### 3.5. Binary Decision Layer

This layer serves as the final classification head of DIFC-Net, transforming the fused feature $f_{acf}\in R^{C}$ from the Active Capture Fusion module into a scalar probability that represents the likelihood of an image being diffusion-generated. Given the unified representation $f_{acf}$, the prediction is obtained through a lightweight linear classifier followed by a sigmoid activation: $\hat{y}=\sigma \left(W_{cls}f_{acf}+b_{cls}\right)$, where $W_{cls}\in R^{1\times C}$ and $b_{cls}\in R$ are learnable parameters, and σ(·) denotes the sigmoid function that maps the output to the range $\left[\begin{array}{c} 0,1 \end{array}\right]$.

The model is optimized under a binary cross-entropy (BCE) objective:

(10) $$L_{BCE}=-\left[y\log\hat{y}+\left(1-y\right)\log\left(1-\hat{y}\right)\right].$$

where y∈{0,1} represents the ground-truth label (0 for real, 1 for fake). This loss penalizes both false positives and false negatives, ensuring stable convergence for imbalanced datasets often encountered in generative-image detection.

Through end-to-end training, the entire network learns to jointly exploit spatial reconstruction cues and temporal diffusion dynamics for robust real-versus-fake discrimination.

## 4. Results

### 4.1. Experimental Setup

#### 4.1.1. Datasets

To evaluate the generalization ability of DIFC-Net, we construct a balanced training set of 60k images, consisting of 30k real samples from LAION [51] and 30k synthetic images generated by Stable Diffusion v1.4. This setup provides a representative in-distribution generator while allowing the model to learn reconstruction- and trajectory-based cues beyond generator-specific artifacts. For fair comparison, all methods, including DIFC-Net and the baselines, are trained on the same Stable Diffusion v1.4 training set and evaluated under the same cross-model protocol.

For evaluation, we adopt an unseen-generator testing protocol in which all test generators are excluded from training. As summarized in Table 2, the evaluation suite includes six diffusion models (DALL·E 3, SDXL, SD-v1.5, ADM, GLIDE, and WuKong), three subsets from MNW [52] (Adobe, Image3, and Baidu), and one GAN model (BigGAN). These generators cover diverse architectures, generation mechanisms, and data sources, thereby providing a broad assessment of cross-model robustness. It should be noted that MNW also contains categories overlapping with generators already included in our main benchmark, such as DALL·E 3 and SDXL. Therefore, we select the non-overlapping MNW subsets so as to avoid duplication and to retain complementary generators that further diversify the evaluation set.

All generator-specific evaluation sets are class-balanced, with equal numbers of real and AI-generated images in each test set. Although the total number of samples varies across generators, this difference is due to data availability rather than class imbalance. In particular, the Adobe, Image3, and Baidu subsets selected from MNW contain 350, 250, and 250 generated images, respectively; accordingly, we use the same numbers of matched real images, resulting in balanced test sets of 350/350, 250/250, and 250/250. For the other generators, the evaluation sets are constructed with 500/500 real/fake samples.

For the real class, we consistently use LAION-based real images as a shared reference pool. This design is intentional: by keeping the real-image distribution fixed across generator-specific test sets, the comparison focuses on differences introduced by the fake generators rather than on source-dependent variations in the real class. In this way, the use of a common LAION real pool reduces dataset bias and provides a more controlled benchmark for evaluating cross-model generalization.

#### 4.1.2. Baselines

To establish comprehensive comparisons with representative previous works, we evaluated DIFC-Net against twelve AIGI detectors spanning spatial, frequency, reconstruction-based, foundation-model-based, and vision–language paradigms. The evaluated baselines include CNNSpot [6], F3Net [32], GramNet [33], UnivFD [34], DIRE [8], SPAI [35], DMID [36], DE-Fake [37], RINE [38], Fusing [39], NPR [40], and C2PClip [41]. As summarized in Table 1, these previous methods cover substantially different detection principles. CNNSpot, F3Net, GramNet, SPAI, DMID, DE-Fake, RINE, Fusing, and NPR mainly rely on image-space artifacts, frequency-domain anomalies, feature statistics, or discriminative forensic representations. UnivFD represents a recent detector design based on large-scale pretrained visual features, while C2PClip further extends this line by leveraging CLIP-based vision–language representations. Among these baselines, DIRE is the most closely related diffusion-oriented detector, as it explicitly exploits inversion and reconstruction inconsistency under diffusion models.

This diversity makes the benchmark particularly challenging and also enables a broad comparison between DIFC-Net and previous works from different methodological families. Unlike prior detectors that mainly depend on static appearance cues, spectral clues, generic pretrained representations, or a single reconstruction-derived signal, DIFC-Net jointly models reconstruction-triplet discrepancy and latent diffusion trajectory with adaptive fusion. Therefore, the empirical comparisons in Section 4.2 evaluate not only whether DIFC-Net achieves competitive detection performance with respect to previous works, but also whether diffusion-intrinsic modeling provides a more robust basis for cross-model AI-generated image detection.

All experiments were conducted on a workstation equipped with an NVIDIA GeForce RTX 4090 GPU (NVIDIA Corporation, Santa Clara, CA, USA). The implementation was based on Python 3.8, PyTorch 2.0, and CUDA 11.8. Diffusion inversion and reconstruction were implemented using the Hugging Face diffusers library, together with the corresponding pretrained Stable Diffusion v1.4 components. Unless otherwise specified, the same software environment was used for all compared methods.

### 4.2. Results and Analysis

#### 4.2.1. Comparisons of Detection

To ensure fairness, all compared methods were implemented and evaluated under the same experimental protocol. In particular, all detectors were trained on the same Stable Diffusion v1.4 training set, validated on the same validation split, and tested on the same unseen generator-specific evaluation sets. The repeated-run analysis and the threshold-dependent metrics were also conducted under the same protocol for all methods unless otherwise specified. Table 3 reports the cross-model detection performance of DIFC-Net and twelve representative previous works on ten unseen generators over five independent runs, where the results are presented as AUC (%, mean ± std). Compared with these previous methods, DIFC-Net is evaluated not only in terms of average detection accuracy, but also in terms of stability across repeated runs and robustness across heterogeneous unseen generators. Therefore, the following analysis further compares DIFC-Net with prior detectors using multi-run statistics, ROC curves, and threshold-dependent metrics, so as to provide a more complete view of its cross-model generalization ability.

From the experimental results, DIFC-Net achieves the strongest average performance across the ten generators, reaching an average AUC of 90.29%. In particular, it attains the best results on DALL·E 3 (98.83 ± 0.51), Image3 (95.41 ± 1.57), and Baidu (87.28 ± 1.30), while remaining highly competitive on SDXL (98.82 ± 0.92), SDv1.5 (99.02 ± 0.61), ADM (85.99 ± 1.20), GLIDE (89.24 ± 1.24), and Wukong (95.41 ± 1.19). Although some baselines achieve the highest score on individual generators, such as C2PClip on SDXL and SDv1.5, GramNet on ADM, RINE on GLIDE and BigGAN, and SPAI on Wukong and Adobe, DIFC-Net exhibits the most balanced behavior across heterogeneous generators, indicating that its effectiveness does not depend on a specific model family or artifact type.

In addition to high mean performance, DIFC-Net also demonstrates strong statistical stability. Its standard deviation remains below 1.30 on eight of the ten generators and does not exceed 1.57 on any generator, which is consistently low compared with many competing methods. This result suggests that the proposed framework is not only accurate, but also robust to run-to-run variation. Such stability is important for cross-model detection, because a detector with strong average performance but large variance may still be unreliable when deployed across unseen generators.

**Figure 3:**
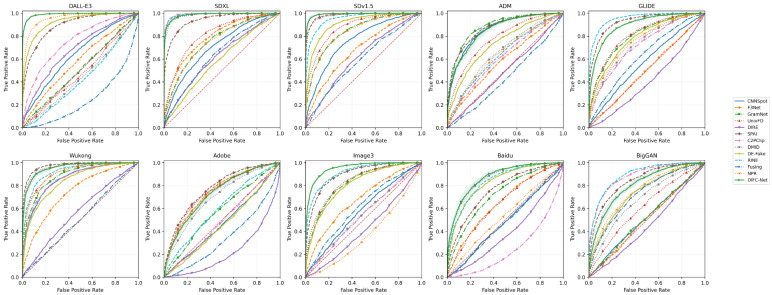
ROC curves of all evaluated methods across ten unseen generators over five independent runs.

Figure 3 further presents the ROC curves of all evaluated methods on all ten generators. The figure provides a more fine-grained view of the cross-model behavior beyond a single scalar metric. On DALL·E 3, SDXL, SDv1.5, Wukong, Image3, and Baidu, the ROC curves of DIFC-Net remain close to the upper-left corner and either outperform or closely track the strongest competing methods, confirming strong separability on both high-fidelity and architecturally diverse generators. On more challenging sources such as ADM, GLIDE, Adobe, and BigGAN, the spread among different methods becomes much larger, which highlights the difficulty of maintaining robust generalization under diverse generation mechanisms. Nevertheless, DIFC-Net still preserves competitive discrimination without severe performance collapse, further supporting the robustness of the proposed diffusion-intrinsic modeling strategy.

Taken together, the evidence from multi-run AUC statistics and generator-wise ROC curves consistently supports the claim that DIFC-Net provides robust cross-model generalization. Its advantage is therefore reflected not only in strong average performance, but also in stable behavior across repeated runs and competitive discrimination over a wide range of unseen generators.

To complement the threshold-independent AUC evaluation, Table 4 further reports the threshold-dependent classification metrics of DIFC-Net, including Precision, Recall, F1-score, and False Positive Rate (FPR). These metrics provide a more direct view of the operating-point behavior of the detector, especially the trade-off between correctly detecting fake images and avoiding false alarms on real images.

DIFC-Net maintains strong threshold-dependent performance on most generators, with particularly favorable results on DALL·E 3, SDXL, and SDv1.5, where both F1-score and FPR indicate highly reliable discrimination. It also remains competitive on Wukong and Image3. By contrast, more challenging generators such as ADM, GLIDE, Baidu, BigGAN, and especially Adobe show a clear degradation in Precision/Recall/F1-score together with increased FPR, suggesting that these sources are more difficult under a fixed decision threshold. Nevertheless, the overall trend still indicates that DIFC-Net preserves a reasonable balance between detection sensitivity and false-alarm control across heterogeneous unseen generators.

**Figure 4:**
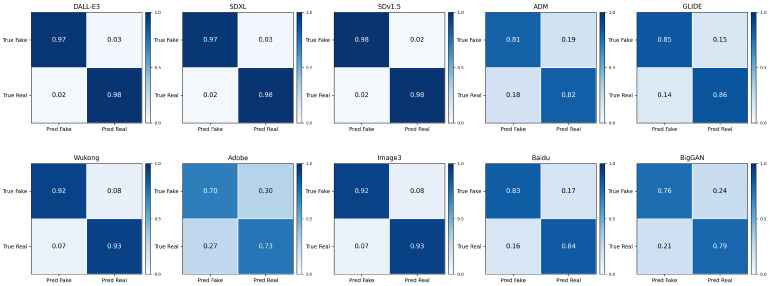
Normalized confusion matrices of DIFC-Net on all evaluated generators. Color intensity reflects the normalized proportion in each cell.

Figure 4 further presents the normalized confusion matrices of DIFC-Net on all evaluated datasets. Consistent with the metrics in Table 4, generators with stronger performance exhibit clear diagonal dominance, while more challenging generators show more pronounced off-diagonal errors. In particular, DALL·E 3, SDXL, and SDv1.5 remain strongly concentrated on the diagonal, whereas Adobe shows the most noticeable confusion between fake and real samples. These results provide a more intuitive view of the detailed error distribution and further demonstrate that DIFC-Net maintains robust operating-point behavior on most unseen generators.

#### 4.2.2. Analysis of Residual and Attention Maps

**Figure 5:**
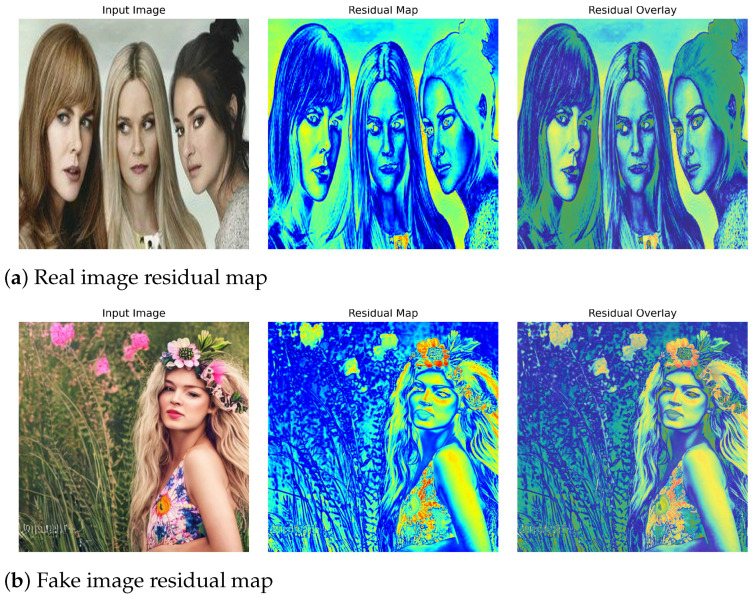
Residual visualization of real and diffusion-generated images. (**a**) Real samples show weak, edge-localized residuals along natural boundaries, indicating consistent reconstruction with the real-image manifold. (**b**) Fake samples exhibit stronger and irregular residual activations over semantic regions, revealing reconstruction inconsistencies effectively captured by DIFC-Net.

To better understand how DIFC-Net differentiates real from diffusion-generated images, we visualize the residual patterns produced during reconstruction (Figure 5). The residual map captures pixel-wise deviations between the input image and its diffusion-based reconstruction, reflecting how well the visual content conforms to the learned natural-image manifold.

In real images, residual responses are weak and spatially coherent. Activations are mainly concentrated along natural high-gradient regions—facial contours, hair edges, and illumination transitions—indicating that the diffusion reconstruction can accurately recover their semantic structure. The low and stable residual energy reflects strong consistency between the input and the diffusion prior.

In contrast, diffusion-generated images show broad and irregular residual activations extending beyond boundaries into semantic regions such as skin, hair textures, and vegetation. These high-magnitude responses reveal inconsistencies in material continuity and lighting statistics—artifacts not present in real imagery. This demonstrates that although synthetic images may appear natural in pixel space, they struggle to remain coherent under reconstruction, producing explicit residual cues that DIFC-Net learns to exploit.

**Figure 6:**
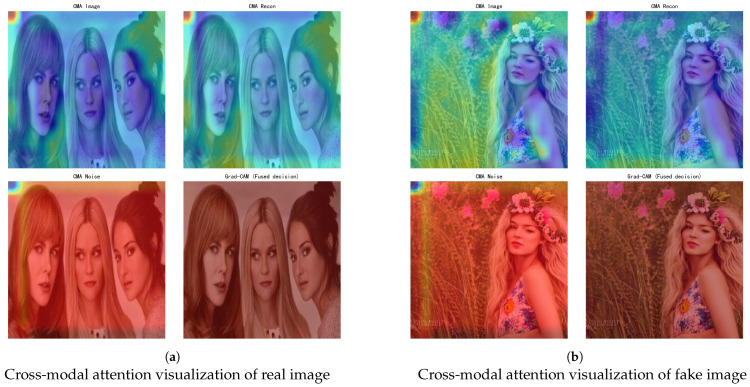
Cross-modal and fused attention visualization of real and diffusion-generated images. (**a**) Real samples exhibit spatially coherent CMA activations focused on object semantics with minimal noise response, yielding compact Grad-CAM decisions. (**b**) Diffusion-generated samples display dispersed, texture-oriented attention with strong noise activation, indicating disrupted cross-modal alignment effectively captured by DIFC-Net.

To further examine how these residual discrepancies influence DIFC-Net’s decision-making, we visualize Cross-Modal Attention (CMA) responses and the fused Grad-CAM activation (Figure 6). While the residual map indicates where reconstruction fails, these attention responses reveal how the network internally attributes such inconsistencies to authenticity prediction.

For real samples, CMA activations are focused and coherent across the image and reconstruction branches, while the noise branch shows negligible activation. The fused Grad-CAM is compact and localized over semantic areas, indicating high agreement between spatial and trajectory features.

For diffusion-generated samples, activations become dispersed and texture-driven. Strong responses appear in synthetic details such as hair strands and floral patterns, and the noise branch becomes highly activated, signaling cross-modal disagreement. The fused Grad-CAM spreads across a wider region, reflecting uncertainty caused by conflicting cues.

#### 4.2.3. Discussion

DIFC-Net detects synthetic images by leveraging a key observation: real and diffusion-generated images behave differently during diffusion inversion. Real images lie close to the diffusion model’s learned manifold, enabling stable reconstruction with low and edge-localized residuals. In contrast, generated images only approximate that manifold in pixel space; when forced through inversion, they cannot maintain latent consistency, producing unstable and spatially dispersed residual activations. This instability reflects a mismatch between synthetic content and the statistical distribution of real images.

The same effect appears in attention responses. Real images yield coherent CMA and Grad-CAM activation concentrated on semantic structures, whereas generated images produce diffuse, texture-driven activations, indicating disagreement across modalities (image–reconstruction–noise).

Thus, DIFC-Net does not rely on visible artifacts or frequency fingerprints. Instead, it judges authenticity based on whether an image remains consistent under diffusion inversion. Residual instability and cross-modal attention disagreement serve as fundamental and model-agnostic evidence for detecting synthetic content.

#### 4.2.4. Computational Complexity and Runtime Analysis

Compared with image-space detectors that directly classify a single input image, DIFC-Net introduces additional computational cost mainly from the diffusion inversion process and the extraction of trajectory-aware representations. Let *T* denote the number of inversion steps. The runtime of the inversion stage grows approximately linearly with *T*, while the subsequent VCP–TFM–ACF feature learning operates on the extracted reconstruction triplet and latent trajectory with fixed network dimensions. Therefore, the overall cost of DIFC-Net is dominated by inversion and can be viewed as O(T) with respect to the number of diffusion steps, given a fixed backbone and feature dimension setting. In our implementation, we adopt T=50 as the default setting, which provides the best trade-off between detection performance and efficiency according to the ablation study.

### 4.3. Ablation Study

To further validate the architectural robustness of DIFC-Net, we conduct an extended ablation study from three aspects: module-wise contribution, fusion strategy, and diffusion inversion step length. All results are reported as average AUC (%, mean ± std) over five independent runs on the ten unseen generators.

The contribution of each architectural component is examined in Table 5. Starting from the raw reconstruction triplet, introducing either VCP or TFM leads to clear performance gains, indicating that both spatial discrepancy reasoning and latent trajectory modeling provide useful detection cues. When the two branches are jointly used, the performance is further improved by a large margin, which confirms their complementarity. After replacing the standard branch combination with the proposed ACF, the average AUC is further increased by 4.78 points, while the variance is reduced by more than half. This suggests that the full framework benefits not only from dual-branch modeling, but also from a more effective and stable integration mechanism.

A comparison of different fusion strategies is reported in Table 6. Simple linear operations, such as element-wise sum and weighted sum, are clearly less effective, implying that direct combination is insufficient to fully exploit the heterogeneous outputs of VCP and TFM. More expressive fusion strategies improve the results, but both concatenation + MLP and cross-attention fusion still remain below ACF. The consistent advantage of ACF over all alternative strategies indicates that its benefit comes from adaptive feature integration rather than from merely increasing fusion complexity.

The sensitivity of DIFC-Net to diffusion inversion step length is summarized in Table 7. Very short inversion trajectories lead to a marked performance drop, suggesting that insufficient diffusion dynamics limit the effectiveness of the detector. As the number of inversion steps increases, the performance improves steadily and reaches its optimum at T=50. Beyond this point, the gain becomes saturated and slightly degrades at longer step lengths. The overall trend indicates that DIFC-Net remains robust within a reasonable range of inversion lengths, while the adopted setting achieves the best balance between effectiveness and stability.

## 5. Conclusions

In this work, we present DIFC-Net, a diffusion-intrinsic framework for AI-generated image detection that moves beyond static pixel artifacts and classifier-driven cues. By explicitly analyzing reconstruction behavior under diffusion inversion, DIFC-Net captures intrinsic discrepancies between real and synthetic images from two complementary perspectives, namely spatial residual inconsistency and latent diffusion dynamics.

The proposed architecture integrates residual comparison, trajectory modeling, and adaptive fusion within a unified diffusion prior, enabling robust and interpretable forensic representation learning. Extensive experiments across diverse generators, including DALL·E 3, SDXL, SD v1.5, ADM, GLIDE, and WuKong, demonstrate that DIFC-Net achieves strong detection performance and favorable cross-model generalization under substantial architectural and distributional shifts. These results suggest that diffusion-intrinsic cues provide a more stable and transferable basis for detecting modern generative imagery, and that jointly modeling reconstruction discrepancies and latent trajectories is crucial for robust detection.

Despite these promising results, several limitations remain. First, DIFC-Net depends on the quality of diffusion inversion, and its effectiveness may be affected when the inversion process becomes less reliable or less stable. Second, compared with lightweight image-space detectors, the inversion-based framework introduces additional computational overhead due to reconstruction and trajectory extraction. Third, although the current experiments demonstrate strong generalization across multiple unseen generators, the evaluation is still limited to the generator families and perturbation settings considered in this work. Therefore, broader validation on more emerging generative models and more challenging real-world degradations is still needed.

Overall, DIFC-Net establishes a reliable and interpretable foundation for diffusion-based image forensics by balancing accuracy, generalization, and transparency. Future work will explore extensions to video and multimodal diffusion models, as well as more efficient designs for scalable and real-time forensic deployment.

## Figures and Tables

**Table 1:** Methodological comparison of representative AI-generated image detection methods and DIFC-Net.

Method	Category	Core Cue	Inv.	Traj.	Key Idea
DIRE [8]	Diffusion-spec.	Reconstruction error after inversion	Yes	No	Detects synthetic images through inversion-induced reconstruction inconsistency.
DRCT [9]	Diffusion-spec.	Contrastive learning on reconstructed views	Yes	No	Learns discriminative representations from reconstruction-based multi-view contrastive training.
DNF [10]	Diffusion-spec.	Noise/residual-based clues	Partial	No	Exploits diffusion noise features and residual cues for detection.
LaRE2 [19]	Diffusion-spec.	Latent reconstruction error	Yes	Limited	Measures latent-space reconstruction inconsistency after inversion.
SeDID [25]	Diffusion-spec.	Reconstruction/noise inconsistency	Yes	No	Uses reconstruction and noise inconsistency as diffusion-specific forensic evidence.
FIRE [26]	Diffusion-spec.	Frequency-guided reconstruction error	Yes	No	Combines reconstruction error with frequency-aware forensic cues.
FakeInversion [27]	Diffusion-spec.	Multi-view inversion features	Yes	No	Fuses original images, decoded noise, and reconstructions as inversion-based multi-view inputs.
TOFE [28]	Diffusion-spec.	Text-guided inversion features	Yes	Limited	Introduces text-conditioned inversion optimization for detection.
DIP [29]	Diffusion-spec.	Reconstruction-consistency fingerprints	Yes	No	Builds source fingerprints from reconstruction consistency of deep image priors.
CNNSpot [6]	General	CNN-learned synthetic artifacts	No	No	Learns generic synthetic-image artifacts with CNN-based classification.
F3Net [32]	General	Frequency-domain artifacts	No	No	Detects manipulations through frequency-domain anomaly modeling.
GramNet [33]	General	Texture/statistical correlations	No	No	Uses texture statistics and Gram-style feature correlations for detection.
UnivFD [34]	Foundation-model	Universal pretrained visual features	No	No	Leverages large-scale pretrained visual representations for universal fake detection.
SPAI [35]	General	Spatial/semantic artifact cues	No	No	Detects AI-generated images through appearance-space inconsistencies.
DMID [36]	General	Deep discriminative artifact cues	No	No	Uses deep discriminative forensic features for synthetic image detection.
DE-Fake [37]	General	Enhanced forensic deep features	No	No	Enhances deep forensic representations for AI-generated image detection.
RINE [38]	General	Robust image forensic features	No	No	Uses robust image forensic representations extracted from intermediate encoder features.
Fusing [39]	General	Global–local feature fusion	No	No	Combines global and local image features for synthetic image detection.
NPR [40]	General	Noise/pattern representations	No	No	Models generic noise-pattern representations as forensic evidence.
C2PClip [41]	Vision–language	CLIP-based contrastive features	No	No	Uses pretrained CLIP representations for contrastive AI-image detection.
DIFC-Net (Ours)	Diffusion-intrinsic	Triplet discrepancy + latent trajectory with adaptive fusion	Yes	Yes	Jointly models reconstruction-triplet discrepancy and latent diffusion trajectory with adaptive fusion.

**Table 2:** Evaluation datasets.

Fake Dataset	Datasize (Fake/Real)	Real Dataset
DALL·E3	500/500	LAION
SDXL	500/500	
SDv1.5	500/500	
ADM	500/500	
GLIDE	500/500	
Wukong	500/500	
Adobe	350/350	
Image3	250/250	
Baidu	250/250	
BigGAN	500/500	

**Table 3:** Cross-model performance on unseen generators over five independent runs. Results are reported as AUC (%, mean ± std). Best in **bold**.

Method	DALL·E3	SDXL	SDv1.5	ADM	GLIDE	Wukong	Adobe	Image3	Baidu	BigGAN	Avg
CNNSpot	63.59 ± 1.42	70.42 ± 1.67	76.08 ± 1.53	85.35 ± 1.63	56.20 ± 0.92	85.78 ± 1.64	45.48 ± 3.19	57.66 ± 1.19	47.63 ± 1.30	51.13 ± 1.12	63.93 ± 1.56
F3Net	57.94 ± 1.87	79.75 ± 1.89	67.97 ± 1.94	56.61 ± 2.19	49.44 ± 1.87	74.99 ± 2.09	73.87 ± 2.21	68.16 ± 2.28	65.17 ± 2.01	49.39 ± 1.86	64.33 ± 2.02
GramNet	51.64 ± 1.89	77.84 ± 2.08	84.26 ± 2.14	**86.72 ± 1.64**	78.53 ± 2.16	88.36 ± 1.71	55.56 ± 2.33	82.69 ± 2.18	70.35 ± 2.32	52.77 ± 2.12	72.87 ± 2.06
UnivFD	47.21 ± 1.64	81.08 ± 1.33	87.98 ± 1.71	77.39 ± 1.29	80.96 ± 1.40	90.92 ± 1.78	75.10 ± 1.81	84.11 ± 1.54	63.40 ± 1.32	61.55 ± 1.34	74.97 ± 1.52
DIRE	69.05 ± 2.03	62.63 ± 2.22	63.02 ± 2.29	49.43 ± 2.20	40.07 ± 2.36	55.64 ± 2.50	24.28 ± 2.58	44.17 ± 2.38	36.51 ± 2.74	44.85 ± 2.24	48.97 ± 2.35
SPAI	90.45 ± 2.06	93.71 ± 2.22	98.19 ± 1.29	85.50 ± 2.67	92.74 ± 2.16	**97.08 ± 1.95**	**78.86 ± 2.27**	90.00 ± 2.23	75.74 ± 2.25	84.83 ± 2.19	88.71 ± 2.13
C2PClip	75.12 ± 1.76	**98.92 ± 0.66**	**99.27 ± 0.65**	60.96 ± 1.61	71.90 ± 1.78	85.02 ± 1.61	49.17 ± 1.66	51.59 ± 1.65	24.61 ± 2.36	89.13 ± 1.48	70.57 ± 1.52
DMID	46.13 ± 1.67	98.21 ± 1.26	97.41 ± 1.73	67.00 ± 2.12	72.55 ± 1.68	95.37 ± 1.60	69.03 ± 1.64	55.58 ± 1.96	85.50 ± 1.87	67.40 ± 2.11	75.42 ± 1.76
DE-Fake	92.53 ± 1.67	60.60 ± 2.42	91.94 ± 1.80	73.10 ± 1.82	78.13 ± 1.61	85.61 ± 1.86	46.91 ± 2.10	81.01 ± 1.73	82.85 ± 1.62	72.47 ± 1.93	76.52 ± 1.86
RINE	41.92 ± 1.77	98.89 ± 1.00	94.94 ± 1.70	66.66 ± 2.35	**96.30 ± 1.64**	92.50 ± 1.67	60.72 ± 1.63	91.60 ± 1.62	84.37 ± 1.59	**89.86 ± 1.61**	81.78 ± 1.66
Fusing	24.97 ± 1.80	64.64 ± 1.63	59.65 ± 1.65	41.68 ± 1.82	60.87 ± 1.93	53.90 ± 2.04	34.96 ± 1.86	60.33 ± 1.89	45.61 ± 2.28	72.65 ± 1.64	51.93 ± 1.85
NPR	96.26 ± 1.47	74.65 ± 1.78	83.65 ± 1.96	64.74 ± 1.37	74.51 ± 1.92	90.26 ± 1.47	73.99 ± 1.58	36.15 ± 1.44	52.38 ± 1.56	74.51 ± 1.90	72.11 ± 1.65
DIFC-Net(Ours)	**98.83 ± 0.51**	98.82 ± 0.92	99.02 ± 0.61	85.99 ± 1.20	89.24 ± 1.24	95.41 ± 1.19	71.63 ± 1.29	**95.41 ± 1.57**	**87.28 ± 1.30**	81.28 ± 1.19	**90.29 ± 1.10**

**Table 4:** Precision, Recall, F1-score, and False Positive Rate (FPR) of DIFC-Net on each generator. Metrics are reported in %.

Dataset	Precision	Recall	F1-Score	FPR
DALL·E3	98.38	97.40	97.89	1.60
SDXL	98.18	97.00	97.59	1.80
SDv1.5	98.40	98.20	98.30	1.60
ADM	81.58	80.60	81.09	18.20
GLIDE	86.00	84.80	85.40	13.80
Wukong	93.13	92.20	92.66	6.80
Adobe	72.19	69.71	70.93	26.86
Image3	93.15	92.40	92.77	6.80
Baidu	83.81	82.80	83.30	16.00
BigGAN	78.56	76.20	77.36	20.80

**Table 5:** Module-wise ablation study of DIFC-Net. Results are reported as average AUC (%) over five independent runs on the ten unseen generators. Best in **bold**.

Variant	Avg. AUC
Triplet Only	46.72 ± 5.94
VCP Only	69.16 ± 2.08
TFM Only	61.55 ± 3.11
VCP + TFM (Concatenation + MLP)	85.51 ± 2.24
DIFC-Net (Full)	**90.29 ± 1.10**

**Table 6:** Comparison of different fusion strategies in DIFC-Net. Results are reported as average AUC (%) over five independent runs on the ten unseen generators. Best in **bold**.

Fusion Strategy	Avg. AUC
Element-wise Sum	74.33 ± 3.18
Weighted Sum	76.06 ± 2.10
Concatenation + MLP	85.51 ± 2.24
Cross-Attention Fusion	82.26 ± 1.85
ACF (Ours)	**90.29 ± 1.10**

**Table 7:** Effect of diffusion inversion step length on DIFC-Net. Results are reported as average AUC (%) over five independent runs on the ten unseen generators. Best in **bold**.

Steps	Avg. AUC
10	68.62 ± 1.98
25	83.47 ± 1.36
50	**90.29 ± 1.10**
100	89.71 ± 1.14
150	89.12 ± 1.42

## Data Availability

Publicly available datasets were analyzed in this study. The corresponding sources are cited in the References section.
